# Decolonizing Science Diplomacy: A Case Study of the Dominican Republic’s COVID-19 Response

**DOI:** 10.3389/frma.2021.637187

**Published:** 2021-03-11

**Authors:** Aída Mencía-Ripley, Robert Paulino-Ramírez, Juan Ariel Jiménez, Odile Camilo

**Affiliations:** ^1^ Research Department, Universidad Iberoamericana, Santo Domingo, Dominican Republic; ^2^ Research Department, Institute for Tropical Medicine and Global Health, Universidad Iberoamericana, Santo Domingo, Dominican Republic; ^3^ Economics Department, Pontificia Universidad Católica Madre y Maestra, Santo Domingo, Dominican Republic; ^4^ Office of the Academic Vice Rector, Universidad Iberoamericana, Santo Domingo, Dominican Republic

**Keywords:** COVID-19, Dominican Republic, decolonial, case studies, higher education managament

## Abstract

The COVID-19 pandemic forced healthcare systems globally to handle a dramatic surge in healthcare utilization while also taxing available testing resources. In the context of healthcare systems in Latin America and the Caribbean, COVID-19 added to the existing burden of infectious diseases related to endemic infections such as arboviruses and HIV. In the Dominican Republic, testing is supplied mostly by the private sector and a national public laboratory. The surge in testing demands laid bare a lack of installed capacities both in laboratory facilities and equipment and trained staff in molecular biology laboratory procedures. This article discusses a case of how science diplomacy and a relatively new law fostering public-private partnerships allowed a university to play a major role in public health response while generating knowledge to inform public policy decisions in an unprecedented manner in the country. Science diplomacy is discussed in the context of decolonization and the importance of the local gaze when creating academic partnerships in the context of global health emergencies.

## Introduction

Broadly defined, science diplomacy allows academic actors to participate via research and other academic endeavors in various state programs and initiatives that generally have a global focus (Ruffini, 2020). In the context of the Madrid Declaration on Science Diplomacy, this is understood as a series of practices in which science, technology, and foreign policy meaningfully interact (Young, 2020). This approach assumes that science diplomacy is more effective than traditional diplomatic approaches because it equally satisfies all involved stakeholders' interests, including governments and non-governmental entities and that the objectives of these efforts have broad political benefits (Young, 2020).

Global issues addressed by science diplomacy can span from environmental issues to global health concerns, and most recently, the Severe Acute Respiratory Coronavirus-2 (SARS-CoV-2), which is the causative agent of the Coronavirus Disease-19 (COVID-19) pandemic. As stated by Young (2020), “the pandemic, like other global challenges, is both knowledge-intensive, in that it requires engagement with scientific knowledge for effective policymaking, and cross-border, in that it is not solvable by a single country acting alone” (p. 1).

In this sense, the COVID-19 pandemic has provided fertile ground for science diplomacy and scientific collaboration in general, as evidenced by the elimination of paywalls to access COVID-19 related data as well as the rapid publication of papers. For example, according to the WHO’s Global research database, as of November 26, 2020, there are 100,562 articles related to COVID-19, probably one of the most researched topics in the timespan of months. These unprecedented collaborative efforts to understand and fight the virus have resulted in articles in which one out of five articles is written by researchers from different countries, fostering what Lee and Haupt (2020) refer to as *scientific globalism*. It should be noted, however, that linguistic barriers for scientists from non English speaking countries remain. Another example of the benefits of science diplomacy is the Vaccine Alliance (GAVI), a partnership that brings together donor and implementing country governments, research agencies, international organizations, vaccine manufacturer and NGOs, which together with the WHO and Coalition for Epidemic Preparedness Innovations lead the COVAX initiative to support the development and manufacture of COVID-19 vaccines (WHO, 2020).

However, developing nations in the Global South and the poorest communities in developed nations have borne the heavy brunt of this disease, with some suggesting it has led to a loss of gains made in achieving the Millenium Development Goals (Mejía et al., 2020), and deviating funding allocated for neglected tropical diseases (Molyneux et al., 2020), and older pandemics afflicting most-at-risk populations like HIV, Tuberculosis, and Malaria (de Souza et al., 2020). According to the United Nations Development Program (UNDP), developing countries are expected to have income losses that would exceed 220 billion, leading to further social and health challenges (IMF, 2020). As such, science diplomacy, when directed by developed nations alone, can replicate colonial structures that continue to ignore the needs of developing nations and silences voices from countries historically ignored by the scientific community. However, science diplomacy continues to assume an equity that breaks with traditional colonialist frameworks that have dominated international research collaborations, especially in global health (Lawrence and Hirsch, 2020).

Science diplomacy requires symmetrical relationships between academic institutions and in-country legal frameworks that can support meaningful collaborations. In the Dominican Republic’s case, where there is low private sector investment in research and development, a new law fostering public-private partnerships allowed an academic institution to work with the state, an international NGO, and an international research laboratory, supported by government entities in both the Dominican Republic and Italy. This multi-sectorial approach allowed for creating a comprehensive COVID-19 response program led by a local university that understood the local context and harnessed community engagement to provide critical data from and by a developing nation. This kind of partnership differs from traditional cooperation initiatives since it encourages and expects local knowledge to inform the methodology, processes, and policy recommendations resulting from the projects, increasing relevance and local ownership of the knowledge that is being produced. The opportunity to participate as equals in global scientific networks is particularly relevant for researchers in developing countries in order to guarantee that the particular needs and challenges will be taken into account.

## Case Study

Universidad Iberoamericana (UNIBE) is a private, not-for-profit university located in the Dominican Republic's capital, with a long-standing tradition of training in health and behavioral sciences. In 2017, UNIBE built the Tropical Medicine and Global Health Institute. The institute's equipment was donated thanks to a competitive grant designed to equip laboratories worldwide, but most specifically those in Low-Middle Income Countries (LMIC). This initiative, Seeding Labs, was created in order to relocate laboratory equipment from mostly north-based universities. Equipment that Seeding Labs allocates to developing countries can be donated by manufacturers as well as universities with surplus equipment. The Seeding Labs initiative has been running for the past 10 years, and for the first time (2017), a local university applied and was granted basic equipment and tools to set up a benchmark laboratory aimed primarily at the study of arthropod-borne diseases like Zika, Chikungunya, and Dengue viruses. This equipment was fundamental in developing a scientific hub and the first of its type in the country, providing the platform for future endeavors, including generating knowledge in case of future epidemics. Seeding Labs and its effort to strengthen developing countries’ research capacities is an example of the emergence of new forms of cooperation in what Appe (2018) describes as a “post-aid world.” The Dominican Republic, as many other Latin American countries, has experienced a significant reduction in aid from traditional donors during the past decades, and as many others like Brazil, Mexico, Colombia and Ecuador is witnessing and promoting civil society-led initiatives based on knowledge transfer, capacity building, and more horizontal relationships.

In the years between obtaining the initial laboratory equipment and the COVID-19 pandemic, the university made a substantial investment in its research infrastructure that included: improvements in human resource policies and compensation to attract and retain researchers, continued investment in infrastructure, creation and improvement of institutional policies and governing bodies related to intellectual property, ethics, and research management, and inserting research across academic disciplines at the university. This last step is critical, as Dominican higher education has historically focused on professional degrees with little research focus. These research specific initiatives were complemented by existing internationalization and continuing education opportunities already available at the University.

By the time the first SARS-CoV-2 infections were detected in the Dominican Republic, the Institute’s molecular biology laboratory had the necessary staff and equipment to join the government's testing efforts. For example, BSL-3 pathogen management training had been conducted in collaboration with a US based university. In addition, prior to the declaration of the state of emergency provoked by the COVID-19 pandemic, the Dominican congress passed a new law providing the legal framework for public-private collaboration in prioritized areas affecting the country. Because of this law, a team of scientists working at the institute identified the equipment, tools, and reagents required to increase diagnostic capacity along with scientific communications and communicated these needs to the government. Effective communication was a crucial aspect of the strategy as the country continues to grapple with pervasive media presence of COVID-19 cures that have no proven efficacy and which are prescribed without regulation (Tapia, 2020). As the university was communicating these needs and developing its strategies, the government was simultaneously working with the private sector in order to obtain more equipment and reagents. Once the public-private collaboration between the university and the government initiated, automatic platforms for rapid diagnosis of SARS-CoV-2 infection in hospitals and community-based settings were set up at the institute and a systematic approach for delivery of results and data analysis was deployed in a record time of three months. This led to the study that sequenced the genome of the country's circulating virus, a collaboration with an international partner in Italy.

## Discussion

The perspectives of science diplomacy from the Global South must be visible in academic literature to provide a complete perspective of the challenges and benefits associated with science diplomacy, especially in global health crises. Sharing these experiences as other Latin American countries like Brazil has done (Riveiro and de Freitas Lima Ventura, 2019) can also foster much needed regional cooperation among Global South countries that may share substantial similarities in terms of legal, healthcare, and academic systems. Several authors (Quadir, 2013; Beleboni, 2019) state that South-South cooperation, which is often knowledge-based, creates conditions for countries to strengthen local capacities and design context-adapted strategies. This model moves away from the conventional, top-down conditionality-driven aid approach and can become a more effective strategy to foster sustainable development.

For these efforts to become sustainable and genuinely address the pressing, systemic problems of the South, the logic of cooperation, regardless of the partners involved, must be transformed. As Chisholm and Steiner-Khamsi (2009) argue, most often, these initiatives, financed by international donors, are conceived in an increasingly standardized and prescriptive environment that leaves scarce room to incorporate the unique perspectives and challenges required for findings to be adequately relevant for the South. The renovated recognition of the importance of science and academia's role in finding solutions to global problems evidenced by COVID-19, is promising in the South, where many universities are working hard to conduct research that informs public policies. Several authors, including Xu (2020), argue that we might be “at the crossroads of our past, present, and future” (p. 29) with regards to redefining future global research, and we add scientific diplomacy.

For low and middle-income countries, investment in research infrastructure is quite onerous, requiring governmental and private collaborations. Every year there are re-emerging or emerging pathogens, global warming adds to these challenges, and as such, systems are constantly under pressure, and health infrastructures exhausted. Provision of investment in basic science research programs, training, *avant-garde* molecular equipment, observatories, and resilient institutions led by the South, provide an answer to future outbreaks and emergencies. This global crisis might be the driver of a new proactive response in which South-South collaboration will be motorized, and the colonial North-South scientific relationship might be revisited.

In order to adequately face current and future health and economic challenges, developing countries need to increase investment in research and development and promote international research cooperation. These are also signaled as basic prerequisite for establishing technology sovereignty, a concept described by Edler and colleagues (2020) as “the ability of a state or a federation of states to provide the technologies it deems critical for its welfare, competitiveness, and ability to act, and to be able to develop these or source them from other economic areas without one-sided structural dependency” (p. 8). Science diplomacy and its underlying principles and procedures to foster initiatives that address global issues, must incorporate clear guidelines in order to promote and preserve technology sovereignty, ensuring sates’ capacities to remain (or become) competitive, provide adequate public services, and efficiently managing current and future crisis.

In the developing world, universities need to play a major role in strengthening international cooperation, including South-South collaboration, as they have historically looked northward for academic mobility, dual degree programs, and research collaborations. They may lead the way by doing more than just providing critical perspectives to this approach, but by actively changing internationalization policies, priorities, and indicators. Creating and increasing participation in research networks and development initiatives based on the principles of mutual learning, collaborative problem solving and co-creation of innovative technologies and expertise, as discussed by Abdenur and Estevão Marques da Fonseca (2013) are some of the mechanisms that researchers and policy makers must promote in order to challenge the current health, economic and social crisis and foster sustainable development.
